# Antimicrobial Susceptibility of Subgingival Bacteria in Canines With Periodontal Disease

**DOI:** 10.1155/vmi/7268343

**Published:** 2026-02-23

**Authors:** Karla Chunga-Quinde, Manuel More-Montoya, Rosario Elera-Ojeda, Marco Guerra-Delgado

**Affiliations:** ^1^ Facultad de Zootecnia, Universidad Nacional de Piura, Piura, Peru

## Abstract

Periodontal disease is a highly prevalent condition in dogs; however, information on subgingival bacterial composition and antimicrobial susceptibility remains limited, particularly in South America. This study evaluated the antimicrobial susceptibility patterns of subgingival bacteria isolated from dogs with periodontitis. Samples were collected from 49 dogs with clinical signs of periodontal disease attending veterinary clinics in Piura, northern Peru. Aerobic, facultative anaerobic, and strict anaerobic bacteria were isolated and identified using culture‐based methods, and antimicrobial susceptibility was assessed by the Kirby–Bauer disk diffusion method against clindamycin, amoxicillin plus clavulanic acid, metronidazole, ciprofloxacin, doxycycline, and cephalexin. A total of 305 pure colonies were isolated, of which 55% corresponded to aerobic and facultative anaerobic bacteria and 45% to strict anaerobes. Eighteen genera of aerobic and facultative anaerobic bacteria and thirteen genera of strict anaerobes were identified, with *Staphylococcus aureus* and *Porphyromonas gingivalis* being the most frequently isolated species. High levels of antimicrobial resistance were observed, particularly to metronidazole (87.9%), while resistance to ciprofloxacin was low (10.49%). Resistance to clindamycin, amoxicillin plus clavulanic acid, doxycycline, and cephalexin ranged between 50% and 70%. These findings highlight the limited effectiveness of commonly prescribed antimicrobials for canine periodontal disease and underscore the risks associated with empirical antibiotic use in veterinary dentistry. The study provides the first regional evidence of antimicrobial resistance patterns in subgingival bacteria from dogs with periodontitis. The results support the need for culture‐guided therapy, antimicrobial susceptibility testing, and the implementation of antimicrobial stewardship strategies in routine veterinary dental practice.

## 1. Introduction

Periodontal disease results from the excessive accumulation of plaque or bacterial biofilm on the tooth surface, initially manifesting as gingivitis and progressing to periodontitis [1, 2]. The disease can be classified into four stages based on the severity of clinical signs and lesions, which are gingivitis, early periodontitis, moderate periodontitis, and advanced periodontitis [3, 4].

Periodontal disease exhibits a high prevalence in dogs [5, 6], which increases with age [7, 8]. Insufficient dental care represents a major risk factor for its development, as noted by Wallis and Holcombe [5], who also highlighted the influence of additional factors, such as genetics, nutrition, and behavior. Also, a higher risk has been reported in brachycephalic breeds compared to mesocephalic breeds [7]. Depending on the degree of severity, periodontal disease can cause halitosis, local infection, tooth loss, pain, weight loss, and behavioral changes and has even been associated with systemic disorders [9–11].

Next‐generation sequencing techniques have enabled the study of the composition of the oral microbiome [12–15] and the identification of influential factors, such as age [16, 17]. The bacteria found in the oral cavity of dogs with periodontal disease have been characterized in different studies [18], where the presence of the genus *Porphyromonas* is frequently reported [19–24]. Polkowska et al. [23] highlighted a higher frequency of *Porphyromonas gingivalis*, *Treponema denticola,* and *Prevotella intermedia*; Kačírová et al. [20] reported the combined presence of *Porphyromonas gulae* and *Tannerella forsythia*. In addition, another study reported a higher frequency of *Peptostreptococcus*, *Actinomyces*, and *Peptostreptococcaceae* in mild periodontitis [25]; the association between the presence of *Treponema* and periodontal disease status has also been reported [4, 26, 27].

Cunha et al. [28] summarized the procedures for the management of periodontal disease, which focus on the prevention and control of dental plaque, and the prevention of local and systemic consequences, and include plaque reduction (mechanical or chemical), dental care actions at home (toothbrushing, specific dental diet, and chew toys), regular periodontal evaluation and veterinary procedures (nonsurgical and surgical), antimicrobial therapy, and other options, such as natural products, host modulation therapy, antimicrobial peptides, photodynamic therapy, probiotics, and barrier dental sealants.

The subgingival biofilm constitutes the primary bacterial reservoir associated with disease progression in dogs with periodontitis [1, 2]. Frequent exposure to antimicrobials, particularly in the context of irrational or empirical use [29, 30], generates sustained selective pressure that promotes the emergence of bacterial populations with increased resistance profiles. Genetic determinants and antimicrobial resistance genes have been reported in canine saliva [31] and dental plaque [12], supporting the role of subgingival bacteria as a relevant reservoir for the development and persistence of antimicrobial resistance.

The emergence of antimicrobial resistance in veterinary medicine is an increasingly common global concern [32, 33]. Within this context, the One Health approach recognizes the importance of antimicrobial resistance surveillance and coordinated action among human, animal, and environmental health sectors, including veterinary medicine [32–35]. Evidence suggesting the potential transmission of resistant bacteria between dogs and humans [36–38] further supports the relevance of antimicrobial susceptibility studies in companion animals.

Some studies have suggested that increasing bacterial resistance may be associated with the frequent and empirical use of antibiotics during periodontal treatment in dogs [29, 30]. Consequently, antimicrobial susceptibility studies focusing on antibiotics commonly used in veterinary dentistry in dogs [29, 30, 39, 40] are essential to guide rational therapeutic decisions and optimize treatment outcomes.

Periodontal disease is also highly prevalent in dogs in Peru [41]; however, there is a lack of local data characterizing the subgingival bacteria associated with canine periodontitis and their antimicrobial susceptibility patterns. This knowledge gap limits evidence‐based antimicrobial selection and stewardship in clinical practice. Accordingly, the objective of this study was to evaluate the antimicrobial susceptibility patterns of subgingival bacteria isolated from dogs with periodontitis, providing the first report of antimicrobial resistance in the study region.

## 2. Materials and Methods

### 2.1. Animals

The study was developed in Piura, located in northern Peru. The inclusion criteria were oral symptoms compatible with periodontitis, adult age, and both sexes. Only patients with the owner’s authorization were included in the study. The patients who were presented with periodontal symptoms, such as halitosis, swollen or bleeding gums, sialorrhea, anorexia, chewing difficulty, and tooth loss, were identified. Exclusion criteria included terminally ill patients, dogs with previous antimicrobial treatment, and patients with concurrent diseases.

Samples were collected from 49 dogs with periodontal symptoms, which were attended to in veterinary centers. Among the total number of dogs, 53% were aged between 3 and 7 years, and 47% were between 7 and 16 years.

### 2.2. Sample Collection

The research was performed with prior authorization from the heads of the veterinary centers and the owners of the patients. The clinical examination and sampling were developed after sedation of the patient. Gingival sulcus depth was assessed by periodontal probing, with values between 0 and 3 mm considered normal [41, 42]. The severity of periodontal disease was classified according to the periodontal stages (2, 3, and 4) as described by Stepaniuk [42].

A swab sample was collected by rubbing the gums and the bottom of the periodontal pockets with sterile wooden swabs, to isolate aerobic and facultative anaerobic bacteria. In addition, another sample was collected by placing sterile paper points under the gum of each tooth for 20 s supported with sterile forceps [43]. For both the canine tooth and the premolar tooth, a sterile paper point No. 30 and a sterile paper point No. 40 were collected, four paper points in total, to isolate strict anaerobic bacteria. The swab samples were transported in tubes with peptone water and the paper point samples in tubes with thioglycolate. The transport of the samples until their processing in the laboratory was performed under refrigerated conditions at 4°C, after their respective identification.

### 2.3. Laboratory Analysis

The bacterial culture from the samples and the antimicrobial susceptibility test were developed in the Veterinary Microbiology Laboratory, Universidad Nacional de Piura. The samples were incubated for 6 h at 37°C. The samples with peptone water, previously incubated, were plated on MacConkey agar, Chapman Stone agar, Baird Parker agar, and blood agar, and were incubated at 37°C for 24 h, with an inverted plate, under aerobic conditions. The samples with thioglycolate, previously incubated, were plated on blood agar and Trypticase Soy agar, and were incubated at 37°C for 15 days, under anaerobic conditions. The colonies were re‐plated to obtain pure cultures. Bacteria were classified and identified to species level based on Gram stain results, macroscopic characteristics of the colonies, enzymatic tests (catalase and oxidase), and biochemical tests (TSI agar and SIM medium).

Antimicrobial susceptibility tests were performed using the Kirby–Bauer agar disk diffusion method. The antibiotics clindamycin, amoxicillin plus clavulanic acid, metronidazole, ciprofloxacin, doxycycline, and cephalexin were evaluated. The antibiotics were selected based on availability and use in the study area. The methodology was performed following the Clinical and Laboratory Standards Institute (CLSI) guidelines [44], and the interpretation of the antibiogram results was based on the measurement of the bacterial growth inhibition zones around the disk with antibiotic as described in the CLSI standard [44].

### 2.4. Statistical Analysis

Associations between bacterial species and antimicrobial resistance profiles were evaluated using the chi‐square test. When statistically significant associations were identified, post hoc analysis was performed using standardized residuals. Bonferroni correction was applied to adjust the significance threshold for multiple comparisons.

### 2.5. Ethical Statement

Sample collection complied with Peruvian National Law No. 30407, “Animal Protection and Welfare Law,” in force in Peru since January 7, 2016. The study protocol was approved by the Director of the College of Veterinary Medicine, since the UNP “Ethics Committee for Scientific Research” (No. 844‐R‐2024, November 4, 2024) was established after the study had been completed.

Owners were provided with information describing the objectives of the study, the type of samples to be collected, and the procedures involved. It was clearly explained that participation in this study was voluntary. All owners had the opportunity to ask questions before providing consent. Owner consent was obtained before any clinical examination or sample collection. All data were anonymized before analysis to ensure confidentiality and privacy.

## 3. Results

The results of isolation and identification of bacteria are shown in Tables 1 and 2. A total of 305 pure colonies were isolated, of which 55% were aerobic and facultative anaerobic bacteria and 45% were strict anaerobic bacteria. According to the results of Gram stain, Gram‐negative aerobic and facultative anaerobic bacteria (35%) and Gram‐negative strict anaerobic bacteria (34%) were the most frequently identified groups, followed by Gram‐positive aerobic and facultative anaerobic bacteria (20%) and Gram‐positive strict anaerobic bacteria (11%). Moreover, this study identified 18 genera of aerobic and facultative anaerobic bacteria and 13 genera of strict anaerobic bacteria.

**TABLE 1:** Aerobic and facultative anaerobic bacteria isolated from dogs with periodontal disease.

Bacteria	Gram stain^a^	Frequency^b^	Percent (%)^c^
*Staphylococcus aureus*	+	39	79.59
*Bacillus sp.*	+	17	34.69
*Enterobacter cloacae*	−	16	32.65
*Proteus mirabilis*	−	15	30.61
*Kluyvera sp.*	−	13	26.53
*Escherichia coli*	−	13	26.53
*Proteus sp.*	−	10	20.41
*Plesiomonas shigelloides*	−	7	14.29
*Aeromonas sp.*	−	7	14.29
*Pseudomonas aeruginosa*	−	6	12.24
*Pasteurella multocida*	−	5	10.20
*Salmonella enterica*	−	5	10.20
*Neisseria sp.*	−	5	10.20
*Actinobacillus sp.*	−	3	6.12
*Lactobacillus sp.*	+	3	6.12
*Citrobacter freundii*	−	2	4.08
*Streptococcus sp.*	+	1	2.04
*Enterococcus sp.*	+	1	2.04

^a^Gram Stain: Gram‐positive (+) and Gram‐negative (−).

^b^The frequency is the number of dogs with bacteria isolated.

^c^The percentage was calculated based on a total of 49 dogs.

**TABLE 2:** Strict anaerobic bacteria isolated from dogs with periodontal disease.

Bacteria	Gram stain^a^	Frequency^b^	Percent (%)^c^
*Porphyromonas gingivalis*	−	26	53.06
*Veillonella sp.*	−	18	36.73
*Capnocytophaga canimorsus*	−	15	30.61
*Prevotella intermedia*	−	15	30.61
*Actinomyces sp.*	+	15	30.61
*Clostridium sp.*	+	13	26.53
*Tannerella forsythia*	−	10	20.41
*Fusobacterium sp.*	−	9	18.37
*Treponema denticola*	−	6	12.24
*Parvicella tangerina*	+	4	8.16
*Eikenella corrodens*	−	4	8.16
*Mycoplasma sp.*	+	1	2.04
*Wolinella sp.*	−	1	2.04

^a^Gram Stain: Gram‐positive (+) and Gram‐negative (−).

^b^The frequency is the number of dogs with bacteria isolated.

^c^The percentage was calculated based on a total of 49 dogs.

The aerobic and facultative anaerobic bacteria with the highest detection rate in patients were *Staphylococcus aureus* (79.59%), *Bacillus sp.* (34.69%), *Enterobacter cloacae* (32.65%), and *Proteus mirabilis* (30.61%) (Table 1). Of the 168 isolated colonies, *S. aureus* had a higher isolation rate (23%), followed by *Bacillus sp.* (10%), *E. cloacae* (10%), and *P. mirabilis* (9%).

The strict anaerobic bacteria with the highest detection rate in patients were *P. gingivalis* (53.06%), *Veillonella sp.* (36.73%), *Capnocytophaga canimorsus* (30.61%), *P. intermedia* (30.61%), and *Actinomyces sp.* (30.61%) (Table 2). Of the 137 isolated colonies, *P. gingivalis* had a higher isolation rate (19%), followed by *Veillonella sp.* (13%), *C. canimorsus* (11%), *P. intermedia* (11%), and *Actinomyces sp.* (11%).

**FIGURE 1:**
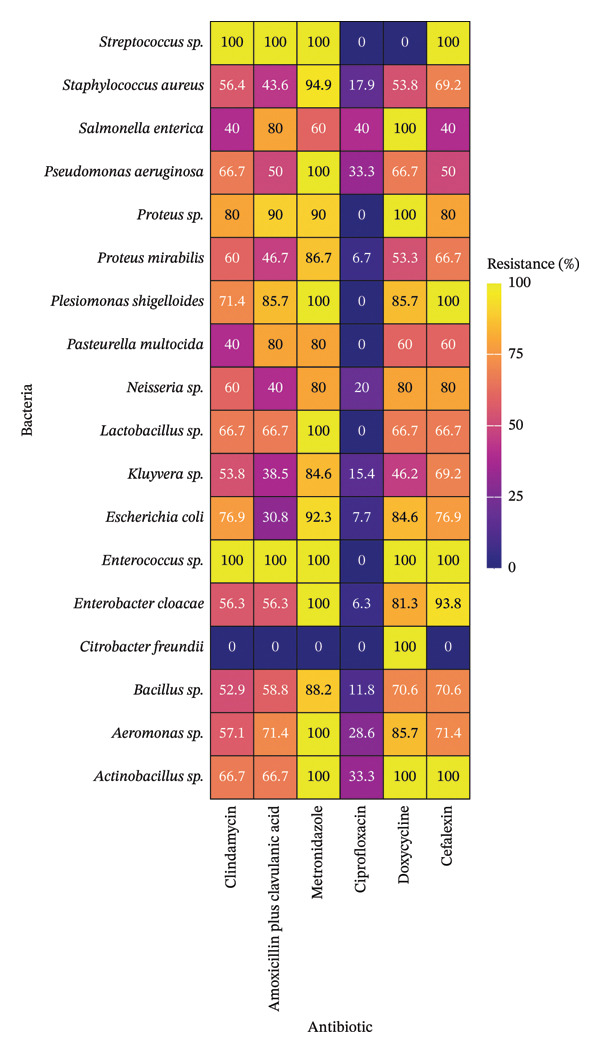
Antimicrobial resistance percentage of aerobic and facultative anaerobic bacteria isolated from dogs with periodontal disease.

The resistance profile of aerobic and facultative anaerobic bacteria to antibiotics is shown in Figure 1. This study identified resistance to metronidazole in 80% or more of the isolated strains for most of the identified bacterial genera (88.9%), including species with the highest rate of isolation. Different levels of resistance were identified in the 89% of the antimicrobial susceptibility results. However, some bacteria showed no resistance, as *Streptococcus sp.* to doxycycline and *Citrobacter freundii* to almost all the antibiotics evaluated. In addition, resistance to ciprofloxacin was 0% in 38.9% of the bacterial genera and reached values of up to 40% in the remaining genera.

The isolated strains of *Streptococcus sp.*, *Pseudomonas aeruginosa*, *Plesiomonas shigelloides*, *Lactobacillus sp.*, *Enterococcus sp.*, *E. cloacae*, *Aeromonas sp.*, and *Actinobacillus sp.* had a resistance to metronidazole of 100%. Likewise, resistance in 100% of the isolated strains of *Streptococcus sp.* and *Enterococcus sp.* to clindamycin and amoxicillin plus clavulanic acid was identified. Resistance to cephalexin was also identified in all strains of *Streptococcus sp.*, *P. shigelloides*, *Enterococcus sp.*, and *Actinobacillus sp.*, while resistance to doxycycline was observed in 100% of the strains of *Salmonella enterica*, *Proteus sp.*, *Enterococcus sp.*, *C. freundii*, and *Actinobacillus sp.*

The resistance to metronidazole was significantly higher compared with other antibiotics for *S. aureus* (*p* value < 0.01) and *E. cloacae* (*p* value < 0.05). Similarly, resistance to doxycycline was significantly greater for *C. freundii* (*p* value < 0.01). In contrast, resistance to ciprofloxacin was significantly lower than that to other antibiotics for *S. aureus*, *Bacillus sp.*, *E. cloacae*, *P. mirabilis*, *Escherichia coli*, *Proteus sp.*, and *P. shigelloides* (*p* value < 0.01).

**FIGURE 2:**
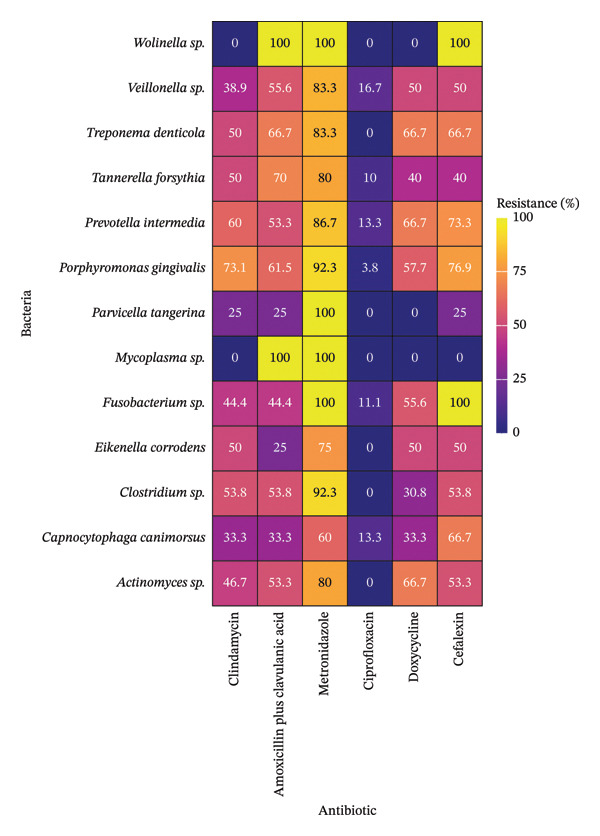
Antimicrobial resistance percentage of strict anaerobic bacteria isolated from dogs with periodontal disease.

The resistance profile of strict anaerobic bacteria to antibiotics is shown in Figure 2. This study identified resistance to metronidazole in 80% or more of the isolated strains for the majority of the identified bacterial genera (84.6%). Different levels of resistance were identified in the 83% of the antimicrobial susceptibility results. However, some bacteria showed no resistance, as *Wolinella sp.* to clindamycin; *Parvicella tangerina* and *Wolinella sp.* to doxycycline; and *Mycoplasma sp.* to four of the antibiotics evaluated. Moreover, resistance to ciprofloxacin was 0% in 53.8% of the bacterial genera and reached values of up to 16.7% in the remaining genera.

The isolated strains of *Wolinella sp.*, *Parvicella tangerina*, *Mycoplasma sp.*, and *Fusobacterium sp.* had a resistance to metronidazole of 100%. In addition, resistance in 100% of the isolated strains of *Wolinella sp.* and *Mycoplasma sp.* to amoxicillin plus clavulanic acid and *Wolinella sp.* and *Fusobacterium sp.* to cephalexin was identified.

The resistance to metronidazole was significantly higher compared with other antibiotics for *P. gingivalis* (*p* value < 0.01), *Veillonella sp.* (*p* value < 0.05), *Clostridium sp.* (*p* value < 0.01), and *Parvicella tangerina* (*p* value < 0.01). In contrast, resistance to ciprofloxacin was significantly lower than that to other antibiotics for *P. gingivalis* (*p* value < 0.01), *Veillonella sp.* (*p* value < 0.05), *P. intermedia* (*p* value < 0.01), *Actinomyces sp.* (*p* value < 0.01), *Clostridium sp.* (*p* value < 0.01), and *Fusobacterium sp.* (*p* value < 0.05).

## 4. Discussion

The bacteria *P. gingivalis* (50%), *E. coli* (40%), and *S. aureus* (30%) were isolated more frequently by Vega et al. [43] in canine patients with moderate‐to‐severe periodontal disease from four shelters. The frequency of *S. aureus* was higher in this study (79.59%), which could be explained using two specific agars for its isolation, Chapman Stone agar and Baird Parker agar. Moreover, the frequency of *P. gingivalis* was similar (53.06%). However, the frequency of *E. coli* was lower (26.53%) in this study compared to the study by Vega et al. [43], where they evaluated canines from shelters; therefore, the increased presence of enteric microorganisms could be due to inadequate sanitary conditions and contamination associated with anal licking and coprophagy.

Polkowska et al. [23] reported a higher detection rate of *P. gingivalis* (100%), *T. denticola* (100%), and *P. intermedia* (55.5%) in a smaller number of canine patients with periodontitis. In addition, Kačírová et al. [20] reported the combined presence of *P. gulae* and *T. forsythia* in a total of seven patients with periodontal disease. In this study, the bacteria *P. gingivalis* (53.06%) and *P. intermedia* (30.61%) had a high frequency in the patients evaluated, but the frequency was lower for *T. forsythia* (20.41%) and *T. denticola* (12.24%). Other studies have evaluated the association between the presence of *Treponema sp.* and the severity of the periodontal disease [4, 26, 27].

*Bacteroides fragilis* had the highest isolation rate (46.1%) of the total anaerobic bacteria isolated in a study reported by Radice et al. [30], followed by *Peptostreptococcus* associated with *P. gingivalis* (30.8%) and *P. intermedia* (23.1%). A higher frequency of *Peptostreptococcus*, *Actinomyces*, and *Peptostreptococcaceae* has also been described in patients with mild periodontitis [25]. Radice et al. [30] also reported a higher isolation rate of *α*‐hemolytic *Streptococcus* associated with *E. coli* (38%) or *Pasteurella multocida* (38%) of the total aerobic bacteria isolated. In this study, *P. gingivalis*, *P. intermedia, Streptococcus*, *P. multocida*, and *E. coli* had an isolation rate of 19%, 10.9%, 0.6%, 3%, and 7.7%, respectively; however, *Bacteroides* and *Peptostreptococcus* were not identified. Another study reported a lower frequency of *P. intermedia* (1.9%) and identified that *Prevotella heparinolytica* was the most frequent (24.1%) of the total *Prevotella sp.* strains isolated [40]. Furthermore, the microbiota in dogs with severe periodontitis after dental extraction has been reported by Šakarnytė et al. [45], and their findings reveal that the most frequent genera were *Porphyromonas* (31.2%), *Prevotella* (9.8%), and *Bacteroides* (8.9%) based on 16S rRNA sequencing data of the microorganisms isolated from samples collected directly from the dental alveolus.

Tavares et al. [46] reported a higher isolation rate of *Neisseria sp.* (31.7%), *Corynebacterium sp.* (12.2%), *Pasteurella sp.* (7.3%), and *Moraxella sp.* (7.3%) in dogs with periodontitis. They also reported that dogs with periodontal disease showed a decrease in *Staphylococcus sp.* compared to healthy dogs. In this research, *Neisseria sp.* and *Pasteurella sp.* had a frequency of 10.20% of the patients evaluated and an isolation rate of 1.6%; however, *Corynebacterium sp.* and *Moraxella sp.* were not identified.

*Porphyromonas sp.* has been reported most frequently in different studies on periodontitis in canine patients [19, 21, 23, 24, 30, 43, 45]. Similarly to this study, *P. gingivalis* was also reported by Radice et al. [30] and Vega et al. [43]; however, Šakarnytė et al. [45] identified that the most prevalent species was *P. gulae* (20.5%). Likewise, Bai et al. [36] reported an isolation rate for *P. gulae* of 31.5% and a detection rate of 92.1% in dogs. Moreover, Khazandi et al. [39] reported an isolation rate of 69.2% for *P. gulae* and 18.5% for *Porphyromonas crevioricanis* in dogs and cats with periodontal disease. In addition, Senhorinho et al. [29] compared the results of bacterial isolation between healthy and sick patients, reporting that *P. gulae* had a higher frequency (82.6%), and *Porphyromonas macacae* had a lower frequency (17.4%) of the total *Porphyromonas sp.* strains isolated from canines with periodontitis; however, they only identified *P. gulae* in canines without periodontitis. Significant enrichment of *P. gulae* in dogs with severe periodontitis was also reported by Niemiec et al. [19]; moreover, the association between the presence of *P. gingivalis/P. gulae* and the severity of the periodontal disease were reported by Takahashi et al. [4]. Other species of *Porphyromonas* have also been described by Stephan et al. [40], who identified that *Porphyromonas circumdentaria*/*Porphyromonas endodontalis* was the most frequent (40.6%), and *P. gingivalis* was the least frequent of the *Porphyromonas sp.* strains isolated (7.7%). Likewise, Wallis et al. [24] identified an increase in the representation of *Porphyromonas canoris* in moderate gingivitis; Hardham et al. [22] described a higher frequency of *Porphyromonas salivosa*, *Porphyromonas denticanis*, and *P. gulae* of the total anaerobic bacteria isolated.

The antibiotics evaluated in this research (clindamycin, amoxicillin plus clavulanic acid, metronidazole, ciprofloxacin, doxycycline, and cephalexin) were selected based on availability and use in the study area. Most of the antibiotics evaluated were reported as the most common antibiotics used in dental procedures that included dental prophylaxis [47, 48] and tooth extraction [48], in canine patients without periodontal disease in veterinary clinics in the United States. These studies reported that cefpodoxime, clindamycin, and amoxicillin plus clavulanic acid had a frequency greater than 10%, followed by cefovecin, amoxicillin, metronidazole, doxycycline, and marbofloxacin with frequencies between 0.5% and 10%. In addition, Weese et al. [48] also included clinical cases of canine patients with periodontal disease and identified that antibiotics were administered to 13% of patients without periodontal disease and 99.9% of patients with different degrees of periodontal disease.

The resistance of *S. aureus* to clindamycin (44.4%), doxycycline (22.2%), cephalexin (22.2%), and amoxicillin plus clavulanic acid (11.1%) was reported by Vega et al. [43]. Moreover, they also reported resistance of *E. coli* to amoxicillin plus clavulanic acid (8.3%), doxycycline (16.7%), ciprofloxacin (16.7%), and cephalexin (8.3%). In this study, *S. aureus* had higher levels of resistance to cephalexin (69.2%), clindamycin (56.4%), doxycycline (53.8%), and amoxicillin plus clavulanic acid (43.6%). Likewise, *E. coli* also had higher levels of resistance to doxycycline (84.6%), cephalexin (76.9%), and amoxicillin plus clavulanic acid (30.8%), but had a lower level for ciprofloxacin (7.7%). Similarly, Radice et al. [30] reported high resistance of *α*‐hemolytic *Streptococcus* associated with *E. coli* to amoxicillin plus clavulanic acid (60%) and doxycycline (80%).

Radice et al. [30] reported resistance of *α*‐hemolytic *Streptococcus* associated with *P. multocida* to amoxicillin plus clavulanic acid (100%) and doxycycline (100%). High resistance levels of *P. multocida* to amoxicillin plus clavulanic acid (80%) and doxycycline (60%) were identified. Likewise, all strains of *Streptococcus sp.* were resistant to amoxicillin plus clavulanic acid in this study but were not resistant to doxycycline.

The resistance of *Neisseria sp.*, *P. mirabilis*, and *P. multocida* to ciprofloxacin and *Streptococcus canis* to doxycycline was reported by Šakarnytė et al. [45]. In this study, strains of *Neisseria sp.* (20%) and *P. mirabilis* (6.7%) resistant to ciprofloxacin were identified, but no resistant strains of *P. multocida* to ciprofloxacin or *Streptococcus sp.* to doxycycline were identified.

This study identified high resistance patterns of *P. gingivalis* to metronidazole (92.3%), cephalexin (76.9%), clindamycin (73.1%), amoxicillin plus clavulanic acid (61.5%), and doxycycline (57.7%), and low resistance patterns to ciprofloxacin (3.8%) compared to other studies. Kim et al. [49] reported no resistance of *Porphyromonas sp.* to amoxicillin plus clavulanic acid and metronidazole, but identified resistance to clindamycin (25%) and ampicillin (17.5%). Radice et al. [30] reported resistance of *P. gingivalis* associated with *Peptostreptococcus* to amoxicillin plus clavulanic acid (25%) and metronidazole (75%). Likewise, Vega et al. [43] reported resistance of *P. gingivalis* to ciprofloxacin (6.7%) and cephalexin (20%); however, they did not identify resistance to the other antibiotics evaluated in this research. The antimicrobial resistance of other *Porphyromonas* species has been evaluated by other studies using the minimum inhibitory concentration (MIC). Resistance of *P. salivosa* to metronidazole was described by Stephan et al. [40], and resistance of *P. crevioricanis* to amoxicillin was reported by Khazandi et al. [39]. Moreover, resistance of *P. gulae* to metronidazole [29] and amoxicillin [39] has also been described.

High resistance patterns of *P. intermedia* to metronidazole (86.7%), cephalexin (73.3%), doxycycline (66.7%), clindamycin (60%), and amoxicillin plus clavulanic acid (53.3%), and low resistance patterns to ciprofloxacin (13.3%) were reported in the present research. Radice et al. [30] reported resistance of this bacterium to amoxicillin plus clavulanic acid (33.3%), doxycycline (66.7%), and metronidazole (100%). Resistance of *P. intermedia* to ciprofloxacin (20%) and cephalexin (40%) has also been described by Vega et al. [43]; however, they did not report resistance to the other antibiotics evaluated in this research. Furthermore, the antimicrobial resistance of *P. heparinolytica* to metronidazole has been identified by Stephan et al. [40] using the MIC.

The findings of this study have clinical relevance, as antimicrobial resistance restricts treatment options, compromises therapeutic efficacy, and increases the risk of clinical failure and disease recurrence [50], ultimately affecting quality of life as well as professional practice and owner confidence. Moreover, this study provides relevant evidence at the public health level, considering the potential exchange of bacteria and genetic determinants of antimicrobial resistance between pets and their owners [51–53].

The high resistance observed to routinely prescribed antibiotics, such as metronidazole, may be associated with the empirical use of antimicrobials, the widespread prophylactic administration in dental procedures [48, 54], and the limited application of microbiological diagnostics and antimicrobial susceptibility testing. These practices generate selective pressure on the microbiome and promote the emergence of resistant bacterial populations.

Within routine veterinary dental practice, these findings highlight the importance of limiting empirical antimicrobial use and prioritizing preventive and diagnostic approaches to periodontal disease management. Preventive strategies, including plaque reduction, home care practices, regular veterinary evaluation and monitoring, and the use of alternative therapies, can reduce the need for antimicrobial treatment [28]. In addition, the prophylactic use of antimicrobials should be restricted to specific clinical indications and patient comorbidities [54, 55], in accordance with the current guidelines.

The identification of bacterial isolates showing low or no resistance to ciprofloxacin suggests that this antimicrobial may remain a viable treatment option in selected cases. Nevertheless, antibiotic administration should be restricted to clinically justified situations and guided by antimicrobial susceptibility testing, and established guidelines for antimicrobial use should be followed. Accordingly, the standardization, implementation, and adherence to antimicrobial stewardship guidelines are essential to promote responsible use [56–60].

The limitations of this study include the lack of molecular confirmation using techniques, such as PCR, metagenomic sequencing, and 16S rRNA gene sequencing [18, 19, 21, 23, 24, 45, 46]. Isolation based solely on microbiological culture methods allows the identification only of microorganisms capable of growing under laboratory conditions, which may lead to an underestimation of microbial diversity [45]. In addition, some bacteria cannot be isolated using conventional culture techniques, require special growth conditions, or are present in very low abundance within the samples, thereby limiting comprehensive characterization of the oral microbiome. Nevertheless, microbiological culture and antimicrobial susceptibility testing based on the disk diffusion method remain widely used in both clinical practice and research settings [52].

This study did not assess associations between bacterial prevalence and disease severity, as the sampling strategy and sample size were not structured to provide balanced representation across all stages of periodontitis. As a result, statistical power for subgroup analysis would have been insufficient.

Variability in the detection of bacterial genera reported in previous studies may be attributable to sample size. Some studies on the oral microbiome and canine periodontitis have been limited by small sample sized [61, 62], which restricts the ability to identify low‐prevalence bacteria that require larger populations for reliable identification. Moreover, the sample size limits statistical inference; therefore, the results should be interpreted as descriptive rather than as definitive estimates of resistance prevalence in other canine populations. In addition, interindividual variability in the oral microbiome related to age, breed, and diet was not evaluated and may have influenced bacterial detection [5, 7, 8].

This study focused on antimicrobial susceptibility in dogs with periodontitis and did not include a healthy control group that would allow comparison of the oral microorganisms between healthy and periodontitis‐affected dogs, as reported in previous studies [19, 25, 46]. Differences in study design have also been noted in these studies: Davis et al. [25] included only mild cases of periodontal disease, Tavares et al. [46] did not specify disease severity, whereas Niemiec et al. evaluated mild, moderate, and severe cases [19]. These variations limit direct comparison across studies.

Future research should incorporate molecular approaches to characterize the oral microbiome and assess the relative abundance of microbial communities, which may influence the presence, pathogenicity, and antimicrobial resistance of specific species. In addition, studies conducted in other geographic regions are needed to evaluate regional resistance patterns and to identify antimicrobial resistance genes and associated genetic determinants. Finally, evaluating the effectiveness of antimicrobial stewardship guidelines, as well as prevention and control strategies for periodontal disease, would contribute to optimizing antimicrobial use in veterinary dentistry.

## 5. Conclusions

*S. aureus* and *P. gingivalis* were the most frequently isolated species in dogs with periodontitis. Subgingival bacteria exhibited heterogeneous antimicrobial resistance patterns to drugs commonly used in periodontal therapy, with predominantly high resistance to metronidazole and low or absent resistance to ciprofloxacin in most isolates.

Antimicrobial resistance is an increasingly prevalent global problem that threatens veterinary practice by reducing the availability of effective therapeutic options. The findings of this study provide clinically relevant evidence on the antimicrobial susceptibility profiles of subgingival bacteria associated with canine periodontitis and underscore the risks associated with continued empirical antimicrobial use in veterinary dentistry.

These results support the need to restrict routine empirical prescription of antimicrobials and to prioritize culture‐based diagnosis and antimicrobial susceptibility testing. Antibiotic use should be limited to cases with clear justification and aligned with antimicrobial stewardship guidelines. Moreover, strengthening preventive strategies to reduce reliance on antimicrobials is essential to mitigate the progression of antimicrobial resistance, with potential implications for both animal and public health.

## Funding

This research received no specific grant from any funding agency in the public, commercial, or not‐for‐profit sectors. The article processing charges (APC) for this publication were funded by Universidad Nacional de Piura.

## Conflicts of Interest

The authors declares no conflicts of interest.

## Data Availability

Data sharing is not applicable to this article. The data generated and analyzed during this study are presented in the current article.

## References

[1] Niemiec B. A. , Periodontal Disease, Topics in Companion Animal Medicine. (2008) 23, no. 2, 72–80, 10.1053/j.tcam.2008.02.003, 2-s2.0-43049181356. 18482707

[2] Harvey C. E. , Management of Periodontal Disease: Understanding the Options, Veterinary Clinics of North America: Small Animal Practice. (2005) 35, no. 4, 819–836, 10.1016/j.cvsm.2005.03.002, 2-s2.0-21044452752. 15979515

[3] Albuquerque C. , Morinha F. , Requicha J. et al., Canine Periodontitis: The Dog as an Important Model for Periodontal Studies, Veterinary Journal. (2012) 191, no. 3, 299–305, 10.1016/j.tvjl.2011.08.017, 2-s2.0-84857235877. 21940182

[4] Takahashi K. , Arima E. , Sawayama H. et al., Possible Correlation of Specific Bacteria Detected by Using a Novel Screening Method and Periodontal Disease in Dogs, Journal of Veterinary Medical Science. (2025) 87, no. 6, 25–0004, 10.1292/jvms.25-0004. PMC12159193 40222921

[5] Wallis C. and Holcombe L. J. , A Review of the Frequency and Impact of Periodontal Disease in Dogs, Journal of Small Animal Practice. (2020) 61, no. 9, 529–540, 10.1111/jsap.13218. 32955734

[6] Butković V. , Šehič M. , Stanin D. , Šimpraga M. , Capak D. , and Kos J. , Dental Diseases in Dogs: A Retrospective Study of Radiological Data, Acta Veterinaria Brno. (2001) 70, no. 2, 203–208, 10.2754/avb200170020203, 2-s2.0-33845900254.

[7] O’Neill D. G. , Mitchell C. E. , Humphrey J. , Church D. B. , Brodbelt D. C. , and Pegram C. , Epidemiology of Periodontal Disease in Dogs in the UK Primary Care Veterinary Setting, Journal of Small Animal Practice. (2021) 62, no. 12, 1051–1061, 10.1111/jsap.13405. 34374104 PMC9291557

[8] Wallis C. , Saito E. K. , Salt C. , Holcombe L. J. , and Desforges N. G. , Association of Periodontal Disease With Breed Size, Breed, Weight, and Age in Pure-Bred Client-Owned Dogs in the United States, Veterinary Journal. (2021) 275, 10.1016/j.tvjl.2021.105717. 34293444

[9] Kurtdede E. , Aralan G. , Cengiz R. S. , Kilinç A. A. , Coşkun Ç. , and Salmanoğlu B. , Evaluation of Systemic Inflammation Parameters in Dogs With Periodontitis, Acta Veterinaria Brno. (2019) 69, no. 2, 218–228, 10.2478/acve-2019-0017, 2-s2.0-85068353774.

[10] Pavlica Z. , Petelin M. , Juntes P. , Eržen D. , Crossley D. A. , and Skalerič U. , Periodontal Disease Burden and Pathological Changes in Organs of Dogs, Journal of Veterinary Dentistry. (2008) 25, no. 2, 97–105, 10.1177/089875640802500210, 2-s2.0-54049125097. 18751659

[11] Nabi S. U. , Wani A. R. , Shah O. S. , and Dey S. , Association of Periodontitis and Chronic Kidney Disease in Dogs, Veterinary World. (2014) 7, no. 6, 403–407, 10.14202/vetworld.2014.403-407, 2-s2.0-84922679141.

[12] Šakarnytė L. , Šiugždinienė R. , Žymantienė J. , and Ruzauskas M. , Comparison of Oral Microbial Composition and Determinants Encoding Antimicrobial Resistance in Dogs and Their Owners, Antibiotics. (2023) 12, no. 10, 10.3390/antibiotics12101554. PMC10604839 37887255

[13] Burgio M. , Pellegrini F. , Frattina L. et al., Oral Microbiota in Cesarean-Delivered Puppies, Frontiers in Veterinary Science. (2025) 12, 10.3389/fvets.2025.1711728. PMC12719267 41438373

[14] Abdolghanizadeh S. , Salmeh E. , Mirzakhani F. , Soroush E. , Siadat S. D. , and Tarashi S. , Microbiota Insights Into Pet Ownership and Human Health, Research in Veterinary Science. (2024) 171, 10.1016/j.rvsc.2024.105220. 38484448

[15] Oba P. M. , Carroll M. Q. , Alexander C. et al., Microbiota Populations in Supragingival Plaque, Subgingival Plaque, and Saliva Habitats of Adult Dogs, Anim Microbiome. (2021) 3, no. 1, 10.1186/s42523-021-00100-9. PMC8130298 34001282

[16] Kislik G. , Zhou L. , Rubbi L. , and Pellegrini M. , Age-Correlated Changes in the Canine Oral Microbiome, Frontiers in Microbiology. (2024) 15, 10.3389/fmicb.2024.1426691. PMC11287893 39081893

[17] Templeton G. B. , Fefer G. , Case B. C. et al., Longitudinal Analysis of Canine Oral Microbiome Using Whole Genome Sequencing in Aging Companion Dogs, Animals. (2023) 13, no. 24, 10.3390/ani13243846. PMC10740535 38136883

[18] Kwack K. H. , Jang E. Y. , Kim C. , Choi Y. S. , Lee J. H. , and Moon J. H. , Porphyromonas Gulae and Canine Periodontal Disease: Current Understanding and Future Directions, Virulence. (2025) 16, no. 1, 10.1080/21505594.2024.2449019. PMC11756583 39834343

[19] Niemiec B. A. , Gawor J. , Tang S. , Prem A. , and Krumbeck J. A. , The Bacteriome of the Oral Cavity in Healthy Dogs and Dogs With Periodontal Disease, American Journal of Veterinary Research. (2022) 83, no. 1, 50–58, 10.2460/ajvr.21.02.0027. 34727048

[20] Kačírová J. , Sondorová M. , Maďari A. et al., Detection of Periodontal Pathogens From Dental Plaques of Dogs With and Without Periodontal Disease, Pathogens. (2022) 11, no. 4, 10.3390/pathogens11040480. PMC9032899 35456155

[21] Santibáñez R. , Rodríguez-Salas C. , Flores-Yáñez C. , Garrido D. , and Thomson P. , Assessment of Changes in the Oral Microbiome that Occur in Dogs with Periodontal Disease, Veterinary Sciences. (2021) 8, no. 12, 10.3390/vetsci8120291. PMC8707289 34941818

[22] Hardham J. , Dreier K. , Wong J. , Sfintescu C. , and Evans R. T. , Pigmented-Anaerobic Bacteria Associated With Canine Periodontitis, Veterinary Microbiology. (2005) 106, no. 1-2, 119–128, 10.1016/j.vetmic.2004.12.018, 2-s2.0-14544274593. 15737481

[23] Polkowska I. , Tymczyna-Borowicz B. , Gołyńska M. , and Nowicka B. , Molecular Microbiological Characteristics of Gingival Pockets in the Periodontal Diseases of Dogs, Journal of Veterinary Research. (2023) 67, no. 1, 115–122, 10.2478/jvetres-2023-0005. 37008776 PMC10062037

[24] Wallis C. , Colyer A. , and Holcombe L. J. , Bacterial Associations With Periodontal Disease in Yorkshire Terriers, BMC Veterinary Research. (2025) 21, no. 1, 10.1186/s12917-025-04541-1. PMC12036286 40295989

[25] Davis I. J. , Wallis C. , Deusch O. et al., Semple M. G. , A Cross-Sectional Survey of Bacterial Species in Plaque From Client Owned Dogs with Healthy Gingiva, Gingivitis or Mild Periodontitis, PLoS One. (2013) 8, no. 12, 10.1371/journal.pone.0083158, 2-s2.0-84892653100. PMC3862762 24349448

[26] Nordhoff M. , Rühe B. , Kellermeier C. et al., Association of Treponema spp. With Canine Periodontitis, Veterinary Microbiology. (2008) 127, no. 3-4, 334–342, 10.1016/j.vetmic.2007.09.011, 2-s2.0-38749086511. 17997236

[27] Kwon D. , Bae K. , Kim H. , Kim S. H. , Lee D. , and Lee J. H. , Treponema Denticola as a Prognostic Biomarker for Periodontitis in Dogs. Brissette CA, PLoS One. (2022) 17, no. 1, 10.1371/journal.pone.0262859. PMC8782364 35061858

[28] Cunha E. , Tavares L. , and Oliveira M. , Revisiting Periodontal Disease in Dogs: How to Manage This New Old Problem?, Antibiotics. (2022) 11, no. 12, 10.3390/antibiotics11121729. PMC9774197 36551385

[29] Senhorinho G. N. A. , Nakano V. , Liu C. , Song Y. , Finegold S. M. , and Avila-Campos M. J. , Occurrence and Antimicrobial Susceptibility of Porphyromonas spp. and Fusobacterium spp. in Dogs With and Without Periodontitis, Anaerobe. (2012) 18, no. 4, 381–385, 10.1016/j.anaerobe.2012.04.008, 2-s2.0-84864375371. 22609780

[30] Radice M. , Martino P. A. , and Reiter A. M. , Evaluation of Subgingival Bacteria in the Dog and Susceptibility to Commonly Used Antibiotics, Journal of Veterinary Dentistry. (2006) 23, no. 4, 219–224, 10.1177/089875640602300404, 2-s2.0-33846528660. 17286127

[31] Tóth A. G. , Tóth D. L. , Remport L. et al., A One Health Approach Metagenomic Study on Antimicrobial Resistance Traits of Canine Saliva, Antibiotics. (2025) 14, no. 5, 10.3390/antibiotics14050433. PMC12108403 40426500

[32] Ruzante J. M. , Harris B. , Plummer P. et al., Surveillance of Antimicrobial Resistance in Veterinary Medicine in the United States: Current Efforts, Challenges, and Opportunities, Frontiers in Veterinary Science. (2022) 9, 10.3389/fvets.2022.1068406. PMC9807758 36605768

[33] Vercelli C. , Gambino G. , Amadori M. , and Re G. , Implications of Veterinary Medicine in the Comprehension and Stewardship of Antimicrobial Resistance Phenomenon. From the Origin Till Nowadays, Vet Anim Sci. (2022) 16, 10.1016/j.vas.2022.100249. PMC9036142 35479515

[34] Cella E. , Giovanetti M. , Benedetti F. et al., Joining Forces Against Antibiotic Resistance: The One Health Solution, Pathogens. (2023) 12, no. 9, 10.3390/pathogens12091074. PMC10535744 37764882

[35] Pattis I. , Weaver L. , Burgess S. , Ussher J. E. , and Dyet K. , Antimicrobial Resistance in New Zealand—A One Health Perspective, Antibiotics. (2022) 11, no. 6, 10.3390/antibiotics11060778. PMC9220317 35740184

[36] Bai Y. , Song P. , Shen Z. et al., Porphyromonas Gulae Infection in Canines, Pet Owners and Veterinarians in China: An Epidemiological Study and Risk Factor Analysis, One Health Advances. (2023) 1, no. 1, 10.1186/s44280-023-00007-x.

[37] Oh C. , Lee K. , Cheong Y. et al., White B. A. , Comparison of the Oral Microbiomes of Canines and Their Owners Using Next-Generation Sequencing, PLoS One. (2015) 10, no. 7, 10.1371/journal.pone.0131468, 2-s2.0-84940030393. PMC4489859 26134411

[38] Tóth A. G. , Tóth I. , Rózsa B. et al., Canine Saliva as a Possible Source of Antimicrobial Resistance Genes, Antibiotics. (2022) 11, no. 11, 10.3390/antibiotics11111490. PMC9686479 36358144

[39] Khazandi M. , Bird P. S. , Owens J. , Wilson G. , Meyer J. N. , and Trott D. J. , In Vitro Efficacy of Cefovecin Against Anaerobic Bacteria Isolated from Subgingival Plaque of Dogs and Cats With Periodontal Disease, Anaerobe. (2014) 28, 104–108, 10.1016/j.anaerobe.2014.06.001, 2-s2.0-84902983405. 24930431

[40] Stephan B. , Greife H. A. , Pridmore A. , and Silley P. , Activity of Pradofloxacin Against Porphyromonas and *Prevotella* spp. Implicated in Periodontal Disease in Dogs: Susceptibility Test Data from a European Multicenter Study, Antimicrobial Agents and Chemotherapy. (2008) 52, no. 6, 2149–2155, 10.1128/AAC.00019-08, 2-s2.0-44449124540. 18411326 PMC2415797

[41] Larraín Y. and Fernández V. , Assessment of the Severity of Periodontal Disease in Upper Premolars Compared to the Lower Premolar Teeth in Canine Patients, Revista de Investigaciones Veterinarias del Perú (RIVEP). (2017) 28, no. 2, 10.15381/rivep.v28i2.13060, 2-s2.0-85028854072.

[42] Stepaniuk K. , Periodontology, Wiggs’s Veterinary Dentistry: Principles and Practice, 2019, Wiley, 81–108, 10.1002/9781118816219.ch5.

[43] Vega H. , Fernández V. , Morales S. , Calle S. , and Pérez C. , Antibiotic *In Vitro* Susceptibility of Subgingival Bacteria in Canines With Moderate to Severe Periodontal Disease, Revista de Investigaciones Veterinarias del Perú (RIVEP). (2014) 25, no. 1, 10.15381/rivep.v25i1.8471.

[44] CLSI , Performance Standards for Antimicrobial Disk Susceptibility Tests; Approved Standard, CLSI Document M02-A11. Eleventh E, 2012, Clinical and Laboratory Standards Institute.

[45] Šakarnytė L. , Mockeliūnas R. , Šiugždinienė R. et al., Microbial Composition of Extracted Dental Alveoli in Dogs With Advanced Periodontitis, Microorganisms. (2024) 12, no. 7, 10.3390/microorganisms12071455. PMC11278955 39065223

[46] Tavares M. de O. , dos Reis L. D. , Lopes W. R. et al., Bacterial Community Associated With Gingivitis and Periodontitis in Dogs, Research in Veterinary Science. (2023) 162, 10.1016/j.rvsc.2023.104962. 37542932

[47] Soltero-Rivera M. , Battersby I. , Morrison J. , Spofford N. , and Weese J. S. , Karobari M. I. , Antimicrobial Use Practices in Canine and Feline Patients With Co-Morbidities Undergoing Dental Procedures in Primary Care Practices in the US, PLoS One. (2024) 19, no. 7, 10.1371/journal.pone.0305533. PMC11236167 38985775

[48] Weese J. S. , Battersby I. , Morrison J. , Spofford N. , and Soltero-Rivera M. , Karobari M. I. , Antimicrobial Use Practices in Canine and Feline Dental Procedures Performed in Primary Care Veterinary Practices in the United States, PLoS One. (2023) 18, no. 12, 10.1371/journal.pone.0295070. PMC10707603 38064486

[49] Kim T. , Choi Y. D. , Hur W. , Lee S. W. , and La T. M. , Antimicrobial Susceptibility of *Porphyromonas* spp. Isolated From Dogs With Periodontal Disease in South Korea, Frontiers in Veterinary Science. (2025) 12, 10.3389/fvets.2025.1684907. PMC12640808 41293225

[50] Chiş A. A. , Rus L. L. , Morgovan C. et al., Microbial Resistance to Antibiotics and Effective Antibiotherapy, Biomedicines. (2022) 10, no. 5, 10.3390/biomedicines10051121. PMC9138529 35625857

[51] Horodyska I. , Kasperska P. , Michalski K. , Bubak J. , Herman I. , and Miszczak M. , Natural Microbiota of Dogs and Cats as a Source and Vector of Resistance Genes—Clinical Significance, International Journal of Molecular Sciences. (2025) 26, no. 16, 10.3390/ijms26167717. PMC12386641 40869035

[52] Casemiro P. A. F. , Ferraz C. M. , Reis M. S. et al., Retrospective Study on Antimicrobial Resistance in Bacteria Isolated From Clinical Infections in Dogs at a Brazilian Veterinary Teaching Hospital, Topics in Companion Animal Medicine. (2025) 68, 10.1016/j.tcam.2025.101004. 40780637

[53] Pieri A. , Aschbacher R. , Fasani G. et al., Country Income is Only One of the Tiles: the Global Journey of Antimicrobial Resistance Among Humans, Animals, and Environment, Antibiotics. (2020) 9, no. 8, 10.3390/antibiotics9080473. PMC7460298 32752276

[54] Volk A. C. , Goldschmidt S. L. , Bollig E. R. , Montebello J. A. , and Granick J. L. , Prophylactic Antibiotic Use is Common in Dogs and Cats Presenting for Procedures at Veterinary Referral Dental Practices, Journal of the American Veterinary Medical Association. (2025) 263, no. 4, 483–491, 10.2460/javma.24.08.0524. 39579477 PMC13220159

[55] Montebello J. A. , Granick J. L. , Bollig E. R. , and Goldschmidt S. L. , Variation in Knowledge, Attitude, and Practices Toward Antibiotic Use Among Diplomates of the American Veterinary Dental College: A Survey-Based Study, Journal of the American Veterinary Medical Association. (2023) 261, no. S2, S6–S13, 10.2460/javma.23.06.0304. 37696501

[56] Richards S. , Bailey K. E. , Scarborough R. et al., Cross Sectional Evaluation of a Large Scale Antimicrobial Stewardship Trial in Australian Companion Animal Practices, Veterinary Record. (2024) 194, no. 4, 10.1002/vetr.3268. 37518680

[57] Feyes E. E. , Diaz-Campos D. , Mollenkopf D. F. et al., Implementation of an Antimicrobial Stewardship Program in a Veterinary Medical Teaching Institution, Journal of the American Veterinary Medical Association. (2021) 258, no. 2, 170–178, 10.2460/javma.258.2.170. 33405979

[58] Farrell S. , Bagcigil A. F. , Chaintoutis S. C. et al., A Multinational Survey of Companion Animal Veterinary Clinicians: How Can Antimicrobial Stewardship Guidelines Be Optimised for the Target Stakeholder?, Veterinary Journal. (2024) 303, 10.1016/j.tvjl.2023.106045. 38000694

[59] Allerton F. , Prior C. , Bagcigil A. et al., Overview and Evaluation of Existing Guidelines for Rational Antimicrobial Use in Small-Animal Veterinary Practice in Europe, Antibiotics. (2021) 10, no. 4, 10.3390/antibiotics10040409. PMC8069046 33918617

[60] Robbins S. N. , Goggs R. , Kraus Malett S. , and Goodman L. , Effect of Institutional Antimicrobial Stewardship Guidelines on Prescription of Critically Important Antimicrobials for Dogs and Cats, Journal of Veterinary Internal Medicine. (2024) 38, no. 3, 1706–1717, 10.1111/jvim.17043. 38465850 PMC11099728

[61] Morita M. , Nambu T. , Yamasaki R. et al., Characterization of Oral Microbiota in 6–8 Month-Old Small Breed Dogs, BMC Veterinary Research. (2024) 20, no. 1, 10.1186/s12917-024-03973-5. PMC10996209 38580990

[62] Wallis C. , Milella L. , Colyer A. , O’Flynn C. , Harris S. , and Holcombe L. J. , Subgingival Microbiota of Dogs With Healthy Gingiva or Early Periodontal Disease From Different Geographical Locations, BMC Veterinary Research. (2021) 17, no. 1, 10.1186/s12917-020-02660-5. PMC7789547 33407419

